# Low Carbohydrate and Low-Fat Diets: What We Don’t Know and Why We Should Know It

**DOI:** 10.3390/nu11112749

**Published:** 2019-11-12

**Authors:** Heather Seid, Michael Rosenbaum

**Affiliations:** 1 Bionutrition Unit, Irving Institute for Clinical and Translational Research, Columbia University, New York, NY 10032, USA; hs3046@cumc.columbia.edu; 2 Department of Pediatrics and Medicine, Division of Molecular Genetics, & Irving Institute for Clinical and Translational Research, Columbia University, New York, NY 10032, USA

**Keywords:** obesity, weight loss, weight gain, diet, fat, carbohydrate, macronutrient

## Abstract

In the 1940s, the diet-heart hypothesis proposed that high dietary saturated fat and cholesterol intake promoted coronary heart disease in “at-risk” individuals. This hypothesis prompted federal recommendations for a low-fat diet for “high risk” patients and as a preventive health measure for everyone except infants. The low carbohydrate diet, first used to treat type 1 diabetes, became a popular obesity therapy with the Atkins diet in the 1970s. Its predicted effectiveness was based largely on the hypothesis that insulin is the causa prima of weight gain and regain via hyperphagia and hypometabolism during and after weight reduction, and therefore reduced carbohydrate intake would promote and sustain weight loss. Based on literature reviews, there are insufficient randomized controlled inpatient studies examining the physiological significance of the mechanisms proposed to support one over the other. Outpatient studies can be confounded by poor diet compliance such that the quality and quantity of the energy intake cannot be ascertained. Many studies also fail to separate macronutrient quantity from quality. Overall, there is no conclusive evidence that the degree of weight loss or the duration of reduced weight maintenance are significantly affected by dietary macronutrient quantity beyond effects attributable to caloric intake. Further work is needed.

## 1. Introduction

According to the Center for Disease Control and Prevention, in 2015–2016, 39.8% of American adults were obese (BMI> 30 Kg/m^2^) [1]. The negative multi-system (health, fat bias, economic) impact of obesity on individual and population health is well-documented [2]. The question remains, is there an optimal diet for weight management and metabolic health?

In 1977, the United States Senate Select Committee on Nutrition and Human Needs presented the Dietary Goals of the United States (the McGovern Report) to the 95th Congress [3]. This report emphasized the health benefits of lower fat higher carbohydrate diets. The USDA partnered with the Department of Health and Human Services to issue the Dietary Guidelines for Americans, which eventually became the UDSA Food Pyramid [4]. These guidelines seem ineffective since the prevalence of obesity and its co-morbidities have continued to increase [5]. Theoretical formulations implicating dysmetabolic consequences of high carbohydrate diets on insulin-adipocyte physiology have resulted in increasing interest in the actively debated hypothesis that obesity and its co-morbidities can be restrained by reducing dietary carbohydrates [6,7,8]. Currently, low carbohydrate and ketogenic diets, once reserved for those managing epilepsy [9], or as treatment for type 1 diabetes [10] prior to the discovery of insulin, are gaining popularity. However, the debate continues over whether a low carbohydrate or low-fat diet is best for preventing weight gain, supporting weight loss, preserving weight maintenance and optimizing cardiovascular and metabolic health.

With the exception of several inpatient studies of the ingestion of diets of varying composition before, during or after weight loss [11], most examinations of the effects of diet composition on weight loss, gain and regain have been outpatient studies in which it is difficult to disassociate physiological effects of a diet from those related to the degree of dietary adherence [6,12,13,14]. The purpose of this manuscript is to evaluate whether or not there is sufficient evidence to conclude that either a low carbohydrate or low fat diet (i.e., relative quantities of fat and carbohydrate as distinct from a discussion of carbohydrate or fat quality) can be recommended as the one most likely to result in sustained weight loss and improved health.

## 2. Methods

It should be noted that this is a narrative review, not a meta-analysis, though numerous meta-analyses are cited. The literature review was performed by searching electronic databases (PubMed and Scopus). The initial search terms with Boolean operators were low carbohydrate AND low fat AND diet AND adult AND clinical trial (*n* = 1573). The search items were then added to create a database that was reviewed for the sections below: energy expenditure (*n* = 564), energy intake (*n* = 603), appetite (*n* = 79), weight (*n* = 972), cardiovascular disease (*n* = 321) and Type 2 diabetes (*n* = 283). There was significant overlap between groups. The articles targeting participants with an underlying disease, such as type 2 diabetes or familial hyperlipidemia, were excluded as these medical conditions could bias the results towards one diet or the other and might not be generalizable to a larger population. Cross-sectional or longitudinal studies of pediatric or geriatric subjects were also excluded. The study methodology was first assessed according to whether the articles were designed to examine the mechanism (e.g., does one diet result in significant changes in variables as predicted) or efficacy (e.g., does one diet promote weight loss, reduced weight maintenance or decreased co-morbidity risk). Studies were also evaluated regarding the duration (e.g., is there adequate time for subjects to accommodate to dietary changes), control (e.g., whether they were conducted on an inpatient or outpatient basis), number of subjects and evidence of adherence. The citations in this manuscript were chosen to illustrate the most relevant data and to be representative of a larger body of work on diet macronutrient balance. The studies targeting specific patient populations with other medical problems that might bias the results of a comparative study, (e.g., type 2 diabetes or familial dyslipidemia) were also excluded. A standard criterion for percent of fat and or carbohydrate prescribed in study diets was not applied as there was no consensus on diet definition in adults at this time. The controlled inpatient studies of longer duration (weeks) or especially informative studies are presented in detail.

## 3. Overview of Low Fat and Low Carbohydrate Diets

### 3.1. Physiological Basis for Low Fat Diet (Figure 1)

**Figure 1:**
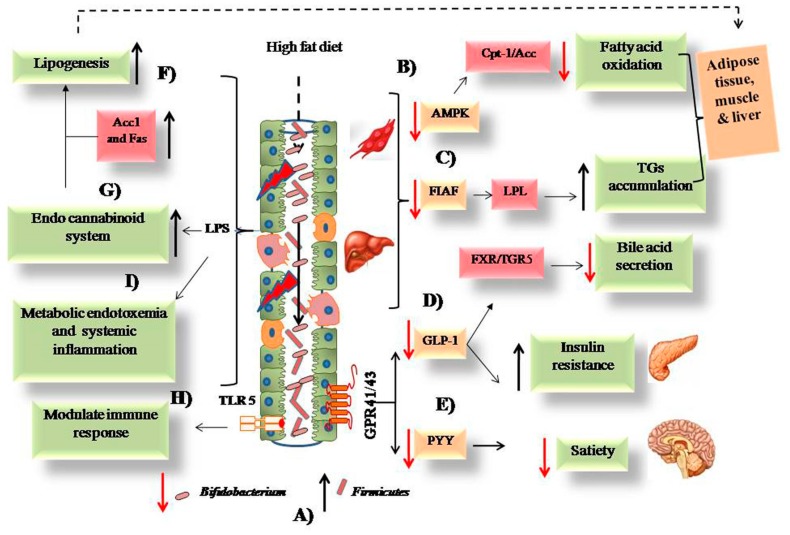
Possible mechanisms associated with the intake of high fat diet and obesity. (A) A high fat diet causes an alteration in intestinal microbiota from low to high Firmicutes and high to low Bifidobacterium. (B) The low expression of adenosine monophosphate kinase (AMPK) leads to decreased fatty acid oxidation. (C) Fasting induce adipose factor (FIAF) expression causes activation of lipoprotein lipase (LPL) that leads to triglyceride (TG) accumulation. (D) Low glucagon-like peptide 1 (GLP-1) leads to increased insulin resistance and decreased bile acid secretion from liver. (E) Decreased peptide YY (PYY) causes low satiety in obese host. (F) Increased lipogenesis via upregulated acetyl-CoA carboxylase (Acc1) and fatty acid synthase (Fas) enzymes. (G) The activation of endo cannabinoid loop via release of lipopolysaccharide (LPS) due to damages intestinal epithelium. (H) The modulation of intestinal immune response via toll-like receptor 5 (TLR-5) downstream signaling. (I) The systemic inflammation caused by inflammatory cytokines and bacterial. (Reprinted from Dahiya et al. [23]). Cpt-1 - carnitine palmitoyltransferase, GPR – G-protein coupled receptors, FXR – Farnesoid X Receptor.

The recommendations for dietary fat restriction arose from the observation that diets high in saturated fat and cholesterol were associated with coronary heart disease [15,16]. Ancel Keys suggested a low-fat diet would help prevent cardiovascular disease [17]. The American Heart Association’s (AHA) subsequent low-fat diet recommendations were also initially intended for those at risk for cardiovascular disease based on family history or their own morbidities [18]. Coupled with the knowledge that fats are more calorically dense than carbohydrates or protein, the AHA recommendations to replace animal fats with non-tropical vegetable oils, also acknowledged the importance of obesity as a risk factor for cardiovascular disease [19]. In 1977, the U.S. Senate’s Select Committee on Nutrition and Human Needs, led by Senator George McGovern, gave a clear government sponsored endorsement for the diet-heart hypothesis [3]. When results from the Framingham Study confirmed an association of obesity and cardiovascular disease risk [20], the diet-heart hypothesis was deemed applicable to adults on the assumption that the lower caloric density and higher thermic effect of nutrients [21] of the low-fat diet would also prevent obesity [22]. Recently, some studies have suggested that high fat diets promote alterations in the gut microbiome (decreased Bifidobacteria and increased Firmicutes) that promote inflammation and decrease satiation [23] (Figure 1). 

### 3.2. Physiological Basis for Low CHO Diet: Insulin-Carbohydrate Model (Figure 2)

The insulin-carbohydrate model is based on the known action of insulin to increase the cellular uptake of glucose and fatty acids, stimulate lipogenesis and inhibit lipolysis [6]. According to this hypothesis, a high carbohydrate diet stimulates insulin release and the resultant decrease in circulating glucose and free fatty acids is then sensed by the central nervous system and other cellular systems regulating energy homeostasis as a state of undernutrition. This invokes subsequent hypometabolism and hyperphagia as well as the preferential storage of ingested calories, such as fat. Clinically, the result is weight/fat gain and increased difficulty in weight management. 

### 3.3. Overview of Energy Balance

In adults, there is remarkable consistency of body weight and composition over time due to a complex interplay of genetic, physiological and behavioral factors [24]. Body weight fluctuates around a set point in a given environment which is influenced by non-homeostatic mechanisms including the hedonic regulation of food intake, and homeostatic mechanisms that regulates the short and long-term energy balance driven by hunger, satiation and changes in adiposity [25]. As an example of the coordination of homeostatic systems regulating energy intake and output, it has been estimated that Americans consume 570 Kcal more per day than 35 years ago, but only 10–20 Kcal per day are stored as additional body weight [25,26,27]. The control of energy stores is achieved through the coordinated regulation of energy intake and expenditure mediated by signals emanating from adipose, gastrointestinal and other endocrine tissues. These signals are then integrated by the liver and by regulatory (hypothalamus, brainstem), hedonic–emotional (amygdala, ventral striatum, orbitofrontal cortex), and executive–restraint (cingulate, middle frontal, supramarginal, precentral, and fusiform gyri) elements of the central nervous system (CNS). Changes in these signals are involuntary and largely due to the reduction in circulating leptin as a result of the loss of fat mass and of the negative energy balance [27]. The consequence is that during and following weight loss, most individuals experience hypometabolism, hyperphagia, neuroendocrine changes (decreased circulation concentrations of bioactive thyroid hormones and leptin) and autonomic changes (decreased sympathetic and increased parasympathetic nervous system tone) that work in concert to favor the return to usual body weight [25,28]. 

These considerations identify the means by which the dietary macronutrient content could meaningfully affect energy balance. Specifically, macronutrient composition would have to either disproportionately decrease appetite and/or increase energy expenditure to promote weight loss and reverse some of the metabolic, behavioral, endocrine and/or autonomic changes that occurred because of weight loss if it is to prevent weight regain. Investigations in this area are further complicated by the possible confounding effects of whether the hypothesis being tested regarding dietary macronutrient balance is examining initial weight gain, weight loss, reduced weight maintenance, or weight regain and whether diets differ in macronutrient quality or quantity. The effects of energy stores versus energy balance on therapeutic results is exemplified by the adipocyte derived hormone leptin. The administration of leptin has little effect on individuals with and without obesity at their usual weight. Leptin repletion has a small effect on appetite but does not affect neuroendocrine function or energy expenditure during weight loss, but at least partially reverses most of the metabolic, behavior, autonomic and neuroendocrine changes that otherwise favor weight regain during reduced weight maintenance [29]. 

## 4. Literature Review

### 4.1. Energy Expenditure

To the authors’ knowledge, there are no long-term studies of the effects of macronutrient balance on energy expenditure during overfeeding. Thearle et al. [30] reported no significant differences in the increase of 24-h energy expenditure above baseline in 20 healthy subjects fed high and low carbohydrate diets for 1 day. Horton et al. [31] studied the effects of 2 weeks of overfeeding with 50% additional calories added as a fat or carbohydrate in 16 healthy inpatient subjects using a crossover design with a 1 week weight maintenance rest period. They found that the increase in energy expenditure above baseline was significantly greater during the high carbohydrate (lower fat) overfeeding than the high fat (lower carbohydrate) overfeeding [31].

**Figure 3:**
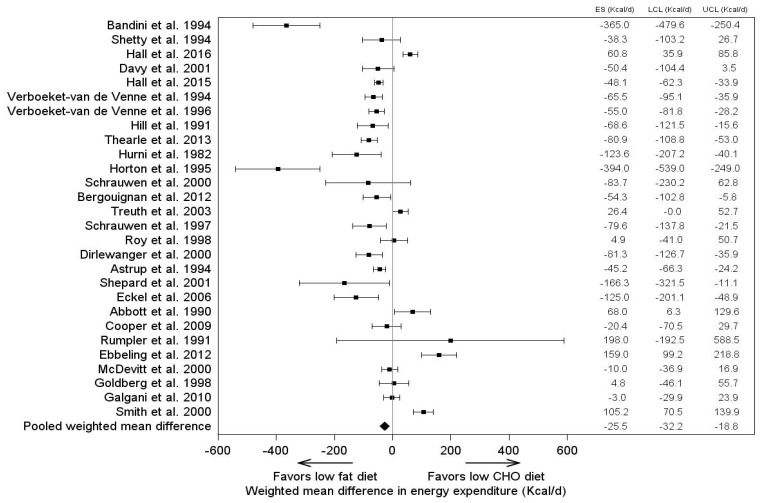
Meta-analysis of the effects of diet low in fat or carbohydrate on energy expenditure in isocaloric studies. Overall, energy expenditure is significantly higher on the low-fat diet (*p* < 0.0001) but the actual value is only 26 Kcal/day. (Reprinted from Hall and Guo [32]).

Outpatient studies comparing the effects on energy expenditure of isocaloric diets with a constant protein intake have yielded varied results. In general, meta-analyses reflect statistically, but probably not physiologically, significantly different dietary fat and carbohydrate effects (Figure 3) [32]. 

In a randomized crossover inpatient study, Leibel et al. [11] reported no effect of a high fat-low carbohydrate diet (70% fat, 15% carbohydrate, 15% protein) versus a low fat-high carbohydrate diet (10% fat, 75% carbohydrate, 15% protein) on weight maintenance and caloric requirements. Hall and colleagues tested the carbohydrate-insulin model of obesity by evaluating the impact of a protein controlled ketogenic diet after a high-carbohydrate baseline diet [33]. In this study, 17 men who were overweight or obese were admitted to an inpatient unit and given an isocaloric diet with a 300 Kcal/day deficit for four weeks (unintentional) [33]. The participants lost weight and body fat, however, the energy expenditure, as measured by whole room calorimetry (57 ± 13 Kcal/day, *p* = 0.030) was found to be physiologically insignificant relative to the overall coupling and day to day variation of energy intake and output as noted in the preceding section regarding energy balance [33]. 

As noted in the Look AHEAD trial [34], and many other studies, only approximately 15% of individuals who successfully and non-surgically lose more than 10% of their usual weight are able to sustain the weight loss [35,36,37,38]. It is, therefore, of great interest whether or not dietary macronutrient balance affects energy expenditure following weight loss, thereby reversing some of the hypometabolism that occurs as a result of weight loss [27]. A 3-way crossover study by Ebbeling et al. evaluated the effect of an isocaloric low-fat diet (LFD) (60% high glycemic carbohydrate, 20% fat, 20% protein), low glycemic index diet (40% carbohydrate, 40% fat, 20% protein) and very low carbohydrate diet (VLCD) (10% carbohydrate, 60% fat, 30% protein) in outpatients with obesity after an initial 10–15% weight loss [13]. Isocaloric diets were administered in randomized order over 4 week periods. The subjects on the VLCD had a 325 Kcal per day greater energy expenditure than the LFD which was dependent somewhat upon the order of testing. Despite the approximately 9100 Kcal greater energy, for the expenditure (i.e., an average of 325 more kcal/day × 28 days) within subjects on the VLCD versus LFD, there was no significant difference in weight loss [13]. The lack of significant weight loss, especially in a within-subjects design study, suggests that the participants under-reported an unapproved intake or engaged in outpatient physical activity that would increase muscle mass during the VLCD as compared to the LFD.

The problem, and potential impact of a lack of control of the subjects’ dietary intake and physical activity in outpatient studies, is illustrated by a second study from the same group evaluating the impact of a high (60%), moderate (40%), or low (20%) carbohydrate diet after weight loss [39]. Again, a change in energy expenditure was found between the participants receiving the low carbohydrate diet compared with those receiving the high carbohydrate diet (209 Kcal per day, CI: 91,326) [39]. This study did not have direct dietary supervision, and the energy expenditure measured by doubly labeled water was over 400 Kcal per day for the high and moderate carbohydrate group and over 600 Kcal per day in the low carbohydrate group when compared against the reported energy intake in the setting of weight maintenance. In addition, the differences between the participants who were in the highest third of pre-weight loss insulin secretion who received the lowest and highest carbohydrate diet was 308 Kcal per day and 478 Kcal per day (*p* < 0.004) [39]. The authors used post-weight loss data, rather than data from an initial weight, as an anchor in these analyses. Approximately 50% of the thermogenic effect of the lowest carbohydrate diet is not seen if baseline data are used. Taken together, these two studies illustrate the ubiquitous problem of an outpatient evaluation of the dietary macronutrient content as a means to examine the mechanisms by which diet manipulations affect energy balance. The potential for the lack of subject compliance, which in and of itself may be affected by diet composition, makes data interpretation extremely difficult [40]. 

It should be noted that dietary protein has been shown to increase energy expenditure. During low fat diet interventions, protein was found to have a slightly positive influence on resting energy expenditure (REE) which accounted for approximately 150 Kcal per day [41]. A meta-analysis of 32 isocaloric controlled feeding studies found that energy expenditure was higher during low fat diet interventions rather than low carbohydrate interventions (26 Kcal per day; *p* < 0.0001) [32]. At this time, however, there is not sufficient evidence consisting of strong inpatient isocaloric feeding studies to support the idea that varying the amounts of carbohydrates or fats in the diet increases energy expenditure and contribute to weight loss and/or weight regain.

### 4.2. Energy Intake: Appetite & Satiety

In order for a tested dietary modification to be a viable lifestyle intervention for obesity management, it needs to be palatable with reasonable adherence. In the short term, after a 14-day low carbohydrate intervention in patients with both obesity and diabetes, no discernable difference in self-reported feelings of hunger, diet satisfaction, or comfort/discomfort levels were found between the study intervention and the participants’ usual diet [42]. A yearlong intervention found overall self-reports of diet satisfaction to be greater with a 40% carbohydrate versus a 50% carbohydrate diet, while a low glycemic index diet was just as satisfying as a high glycemic index diet [43]. Additionally, the appetite reducing hormone pancreatic peptide YY was found to be more reduced in the participants receiving a low-fat diet than a low carbohydrate diet. However, there was no difference in ghrelin levels or self-reported appetite [44]. During weight loss, low carbohydrate diets have been shown to have better compliance but this is not continued after the initial weight loss phase [45]. Other long-term studies (≥1 year) have not shown a difference between low carbohydrate and low-fat diets in regards to intake [46,47]. 

Fat is an important modulator of perceived diet satisfaction and patients given a high fat diet (as percent of total calories) interestingly reported a higher desire for sweetness when compared to a control group [48]. To complicate the issue, both active weight loss and static reduced weight maintenance (i.e., energy balance and energy stores) affect appetite and satiety. During caloric restriction, appetite is increased [49]. After an 8-week weight loss period, overweight and obese participants reported a decreased desire to eat, decreased hunger and increased fullness [50]. In other studies, participants reported an increased appetite and desire to eat with decreased fullness following weight reduction [51,52]. 

A meta-analysis of the impact of energy restricted ketogenic low carbohydrate diets (KLCD) on subjective appetite (visual analogue scale) ratings in three studies found that the KLCD resulted in small but significant differences in hunger and the desire to eat during energy restriction [53], i.e., the KLCD did not result in the anticipated increase in hunger during energy restriction. It is unclear whether these effects were due to ketosis or other aspects of the KLCD. In a 12-week ketogenic diet intervention study, the participants with obesity experienced decreased appetite and decreased reports of emotional eating, but again this was in the setting of significant weight loss (−18 ± 9 Kg men versus −11 ± 3 Kg women; *p* < 0.001) [54]. 

Though the effect of the ketogenic diet on appetite is small, it should be considered in the context of a number of studies reporting greater initial weight loss on a low carbohydrate versus a low-fat energy restricted diet without a significant effect on weight regain as discussed below. The possible effects of dietary macronutrient content on appetite—either before, during or after weight loss— raises the issue of dietary adherence, which is a key component of success of any nutritional therapeutic regimen. In the Dietary Intervention Randomized Control Trial (DIRECT), there was initially greater adherence to a low carbohydrate or Mediterranean diet than a low-fat diet during the first 5 months of a 6-month weight loss phase [45]. However, this did not persist into the weight maintenance phase and, as discussed below, it is not reliably reflected in greater weight loss on low carbohydrate diets.

### 4.3. Weight Management

Weight gain or loss, and the ability to sustain weight loss, is a key metric for evaluating the efficacy of a dietary change. Correlational analyses of dietary fat and carbohydrate content with weight gain have suggested that increases in either are associated with an increased prevalence of obesity, which is presumably due to the ingestion of more calories [55,56,57]. There are, to the authors’ knowledge, no long-term studies of the effects of a prolonged controlled overfeeding of diets with a different macronutrient balance on weight gain, though a recent meta-analysis of 22 studies on obesity-risk in high versus low carbohydrate diets reported no differences [58]. The data regarding weight loss during caloric restriction on low fat versus low carbohydrate diets are varied [55]. Some studies reported greater weight loss on a low carbohydrate diet [59], or low-fat diet [60] while others reported no significant differences [46,61]. Meta-analyses reported statistically, but not medically, significant differences in weight loss (Table 1).

The data regarding the long-term success of low fat and low carbohydrate diets are inconclusive [66]. Foster et al. [59] followed the subjects during and after a comprehensive lifestyle modification program over a 2-year period and did not find differences in weight loss or weight regain (reduced weight maintenance). As shown in Table 2, the data analyzed from meta-analyses remain inconclusive as to whether low carbohydrate or low-fat diets are more associated with prolonged reduced weight maintenance. 

Numerous randomized controlled trials have illustrated that individuals can effectively lose weight on calorie-reduced diets that are either low carbohydrate or low fat [63]. However, obesity and associated health risks present a long-term challenge for management. The accuracy of long-term feeding studies is difficult, if not impossible, to assure in outpatient settings. Even in outpatient studies where participants were able to choose the diet they followed, the autonomy did not improve long-term weight loss [74]. 

### 4.4. Diabetes and Cardiovascular Disease Risk 

Both low carbohydrate and low-fat diets have been studied extensively in regards to mediating cardiovascular disease (CVD) risk. Overall, these studies reveal discrete benefits to both diets but probably no significant differences in the overall health assuming similar carbohydrate and fat quality if not quantity. In a meta-analysis, Sackner-Bernstein et al. reported that a low carbohydrate diet was associated a significantly greater weight loss (~2Kg), and lower predicted risk of cardiovascular disease events based on lower triglycerides (−29.7 mg/dL) and systolic blood pressure (−2.3mm) but higher LDL (9.1 mg/dL) and total (0.6 mg/dL) cholesterol [62]. A more recent meta-analyses from the National Lipid Association Nutrition and Lifestyle Task Force concluded low carbohydrate and very low carbohydrate diets are not superior to other dietary approaches for weight loss, may be more beneficial than low fat diet in reducing triglycerides, with mixed reported effects on LDL cholesterol, and no effects on other cardiometabolic disease markers [75]. 

Gardner et al. [46] found no significant effects of healthy low fat versus low carbohydrate diets on insulin release at 30 min during a glucose tolerance test. Rosenbaum et al. [76] performed biochemical analyses of the inpatient control versus a ketogenic diet study in 17 men described above [33] and found that a ketogenic diet was associated with decreased insulin sensitivity during a control or ketogenic mixed meal tolerance test. However, fasting insulin sensitivity measures (HOMA-IR) indicated that insulin sensitivity improved during the ketogenic diet. In the same study, the findings regarding cholesterol were similar to those reported in meta-analyses (increased total and LDL cholesterol and decreased triglycerides on the ketogenic diet) [76]. 

### 4.5. Other Relevant Considerations

There are a number of issues relevant to the topic of low carbohydrate versus low fat diets that are beyond the purview of this article, but should be briefly discussed. These relate to the patient populations being studied [75,77,78,79,80] and to the dietary macronutrient quality versus quantity [81,82,83,84]. It should be noted, however, that there is evidence regarding cardiometabolic and endocrine health effects of dietary macronutrients in individuals with type 2 diabetes. 

This review focused on the relative quantity of dietary fat and carbohydrate without a discussion of quality. A number of studies suggested that food processing, fiber content, carbohydrate complexity, fatty acid quality, etc., may exert independent effects on appetitive, adiposity-related co-morbidity risk, etc. Ultra-processed foods, which make up a large portion of the American food system, are associated with weight gain and an increased obesity risk [81]. Aside from the calories in, calories out aphorism, the type and quality of calories consumed does impact weight status and disease risk [82]. Future studies evaluating the impact of macronutrient quantity should also evaluate the quality, as they are not mutually exclusive. For example, an international panel of experts conducted an updated meta-analysis and concluded that higher glycemic index and glycemic load carbohydrate were associated with an increased diabetes risk [83]. However, a similar study found an overall 15–30% decrease in cardiovascular disease risk for individuals on a high versus low fiber diet [84]. Diets that are low in carbohydrate but high in unsaturated fat have shown greater improvements in blood glucose stability when compared to high carbohydrate-low fat diets [80].

The results from the studies and meta-analyses of large populations cannot necessarily be extrapolated to individuals with specific medical conditions or risks, such as the degree of effective glucose homeostasis, which were, as noted in the Methods, not emphasized in this review. Hjorth et al. [85] reported that individuals with pre-diabetes and a high fasting insulin lost more weight on a low-fat diet, while those with lower fasting insulin were more responsive to a low carbohydrate diet. Other studies have indicated that low carbohydrate diets are beneficial in the control of diabetes [86]. Specifically, when compared to a standard low-calorie diet, a ketogenic low carbohydrate diet appears to improve glycemic control by reducing fasting glucose and glycosylated hemoglobin levels [77]. Additionally, low carbohydrate diets have been shown to decrease antidiabetic medication requirements [75,79]. Again, however, the effect of the amounts of macronutrients provisioned may be complicated by the weight loss achieved on a calorie-restricted diet as well as by diets varying in the quality versus the quantity of fats and carbohydrate. On the other hand, Gardner et al. [46] found no effects of type 2 diabetes risk allelic variants or circulating insulin concentrations in response to low carbohydrate or low-fat diets. Clearly, more investigation is needed to leverage both current and future treatments for obesity and its co-morbidities based on individual phenotypes and genotypes.

## 5. Discussion

The major conclusion of this review is that there is insufficient evidence to conclude that a low carbohydrate or low-fat diet is superior as a means to prevent obesity or to achieve or maintain weight loss. The lack of controlled prolonged inpatient studies raises uncertainties as to whether the mechanistic consequences predicted from these diets (e.g., increased energy expenditure and decreased appetite on a low carbohydrate diet) are of physiological significance. The difficulty in dietary supervision raises uncertainties regarding any study comparing diet efficacy on an outpatient basis unless compliance can be accurately assessed and included as a covariate in the analysis.

A review of the literature regarding low fat and low carbohydrate diets is complicated by the fact that currently there are no standard definitions of these diets. The American Academy of Family Physicians has defined a low carbohydrate diet as having <20% of calories from carbohydrate [87]. The Atkins diet that was popularized in the 1970s restricts carbohydrate intake to 15–20g per day during the induction phase [88]. Whereas ketogenic diets encourage 90% of calories to come from fat, 1g/Kg of protein and minimal (<15g per day) carbohydrates [89]. The traditional ketogenic diet has a ratio of fats to protein and carbohydrate of 4:1, but there have been numerous adjustments to this with the development of diet offshoots, such as modified Atkins and low glycemic index diet [89]. Similarly, there is not a standard low-fat diet definition, but generally <30% Kcal from fat can be considered a low-fat diet and <20% a very low-fat diet [90]. Without standard definitions, the idea that these diets could become universally prescriptive under any circumstances is unlikely. 

Highly controlled and precise diet studies are inherently difficult to execute. Outpatient studies lack the required oversite to ensure that dietary interventions are appropriately followed and intakes are accurately reported. Dietary self-reports have an important role in epidemiological and nutrition research, however, they should not be used as a measure of true energy intake or for determining diet-health associations [91]. The errors in exercise and activity self-reports also have the potential for introducing error in energy balance studies. At this time, large-scale inpatient trials of low carbohydrate and low-fat diets are lacking in the literature. However, even when executed to perfection (and often at great expense), inpatient interventions do not always have feasible real-world applications.

## 6. Conclusions

In conclusion, at this time, the current evidences do not strongly favor low carbohydrate or low-fat diets for weight management. Future precision medicine studies may permit the identification of specific phenotypes or genotypes that may indicate best practices for the appropriate use of a dietary macronutrient content (both quality and quantity) as therapy for the prevention or treatment of obesity. In the meantime, making general population-level diet recommendations with insufficient evidence should be avoided. 

## Figures and Tables

**Figure 2:**
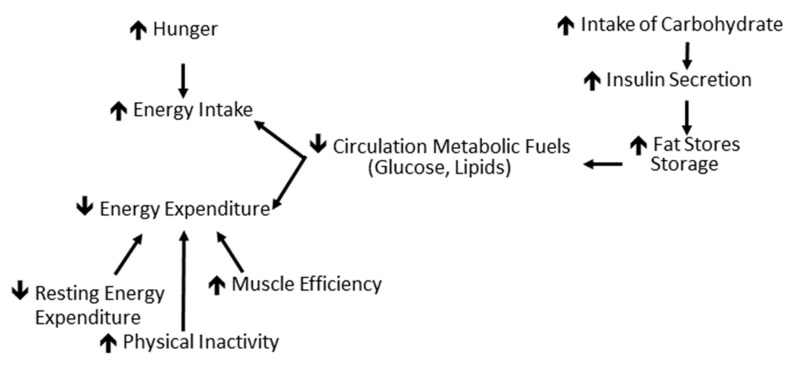
Schematic of the insulin-carbohydrate model. Increased carbohydrate intake promotes increased insulin secretion resulting in depletion of circulating concentrations of metabolic fuels that are used in lipogenesis. The decreased circulating glucose and lipids results in adaptive thermogenesis and hyperphagia which promote weight gain or regain (Based on Ludwig [6]).

**Table 1:** Summary of meta-analyses of the effects of low-carbohydrate versus low-fat weight-loss diets. LC—low carbohydrate, LF—low fat, NS- not significant. # Studies Cited—Number of studies cited in the meta-analysis.

Reference	# Studies Cited	Comparisons	Conclusions
Hall & Guo, 2017 [32]	32	LC vs LF	16 g/day greater fat loss on LF (*p* < 0.001)
Sackner-Bernstein, et al., 2015 [62]	17	LC vs LF	2.0 Kg greater weight loss on LC (*p* < 0.001)
Johnston, et al., 2014 [63]	48	LC vs LF	No significant difference at 6 or 12 mos.
Gow, et al., 2014 [5]	7	LC vs LF	No significant difference
Bueno, *et al.,* 2013 [64]	12	LC vs LF	0.91 Kg greater reduced weight maintenance at least 12 months out on LC (*p* = 0.042), NS at 24 months
Hu, et al., *2015* [65]	23	LC vs LF	1.0 Kg greater weight loss on LC (NS)

**Table 2:** Summary of meta-analyses of the effects of diet macronutrient content on reduced weight maintenance based on Fogelholm et al. [67]. LC; low carbohydrate, HP; high protein, LP; low protein, HF; high fat, LF; low fat, GI; glycemic index.

Diet Meta-Analyses	# Subjects	Conclusion
LC/HP vs. HC/LP [68,69]	120	Inclusive
LC/HP vs. Control [70,71]	973	Inclusive
LC/HF vs. Control [71]	77	Inclusive
LC/HF vs. HC/LF [71]	99	Inclusive
HC/LF vs. Control [71,72]	175	Inclusive
Low GI vs. High GI [73]	773	Inclusive
